# Structure, Dynamics, and Ligand Recognition of Human-Specific CHRFAM7A (Dupα7) Nicotinic Receptor Linked to Neuropsychiatric Disorders

**DOI:** 10.3390/ijms22115466

**Published:** 2021-05-22

**Authors:** Danlin Liu, João V. de Souza, Ayaz Ahmad, Agnieszka K. Bronowska

**Affiliations:** 1 Chemistry—School of Natural and Environmental Sciences, Newcastle University, Newcastle NE1 7RU, UK; D.Liu12@newcastle.ac.uk (D.L.); j.v.de-souza-cunha2@newcastle.ac.uk (J.V.d.S.); A.Ahmad2@newcastle.ac.uk (A.A.); 2 Newcastle University Centre for Cancer, Newcastle University, Newcastle NE1 7RU, UK

**Keywords:** α7 nicotinic receptors, CHRNA7, CHRFAM7A, molecular dynamics, umbrella simulations, coarse grain simulation

## Abstract

Cholinergic α7 nicotinic receptors encoded by the *CHRNA7* gene are ligand-gated ion channels directly related to memory and immunomodulation. Exons 5–7 in *CHRNA7* can be duplicated and fused to exons A-E of *FAR7a*, resulting in a hybrid gene known as *CHRFAM7A*, unique to humans. Its product, denoted herein as Dupα7, is a truncated subunit where the N-terminal 146 residues of the ligand binding domain of the α7 receptor have been replaced by 27 residues from FAM7. Dupα7 negatively affects the functioning of α7 receptors associated with neurological disorders, including Alzheimer’s diseases and schizophrenia. However, the stoichiometry for the α7 nicotinic receptor containing dupα7 monomers remains unknown. In this work, we developed computational models of all possible combinations of wild-type α7 and dupα7 pentamers and evaluated their stability via atomistic molecular dynamics and coarse-grain simulations. We assessed the effect of dupα7 subunits on the Ca^2+^ conductance using free energy calculations. We showed that receptors comprising of four or more dupα7 subunits are not stable enough to constitute a functional ion channel. We also showed that models with dupα7/α7 interfaces are more stable and are less detrimental for the ion conductance in comparison to dupα7/dupα7 interfaces. Based on these models, we used protein–protein docking to evaluate how such interfaces would interact with an antagonist, α-bungarotoxin, and amyloid Aβ_42_. Our findings show that the optimal stoichiometry of dupα7/α7 functional pentamers should be no more than three dupα7 monomers, in favour of a dupα7/α7 interface in comparison to a homodimer dupα7/dupα7 interface. We also showed that receptors bearing dupα7 subunits are less sensitive to Aβ_42_ effects, which may shed light on the translational gap reported for strategies focused on nicotinic receptors in ‘Alzheimer’s disease research.

## 1. Introduction

Nicotinic α7 receptors, encoded by the *CHRNA7* gene, are ligand-gated ion channels involved in cognition, memory, and immunomodulation, which have emerged as attractive targets for neuropsychiatric disorders, neuroinflammation, neuropathic pain, and autoimmune diseases [1,2]. The overall three-dimensional structure of nicotinic α7 receptors is well characterised [3]. Each receptor comprises five identical α7 subunits that are symmetrically arranged around the central ‘channel’s axis. Each subunit contains a large agonist-binding extracellular domain, a transmembrane region formed by four helices, and an intracellular domain-containing receptor modulation and protein–protein interactions [4]. The acetylcholine (ACh) binding sites are located at the interfaces of the extracellular domains of adjacent subunits. The α7 receptor has five identical ACh binding sites; however, ACh binding to only one site is sufficient for the receptor activation [5]. ACh binding triggers the conformational changes that propagate from the binding site toward the interface between the extracellular and transmembrane domains, called the coupling region. These conformational changes contribute to the molecular mechanism of receptor activation [5]. The secondary structure of each subunit comprises a long hydrophilic extracellular domain (EC domain) with an N-terminal β-sheet, three hydrophobic transmembrane regions (M1–M3) that are all α-helical (TM domain), followed by an intracellular loop (IC domain), a fourth transmembrane helix (M4), and an extracellular C-terminus [3,6,7,8]. Upon agonist binding to the extracellular domain, all subunits undergo a significant conformational change, which results in the opening of the transmembrane hydrophilic channel. Hallmarks of α7 receptors include their high Ca^2+^ permeability and very rapid desensitisation [5].

In humans, exons 5–10 in *CHRNA7* may be duplicated and fused to exons A–E of *FAM7A* (family with sequence similarity 7A), resulting in the hybrid gene denoted as *CHRFAM7A* [9,10,11]. Its product, denoted as Dupα7, is a truncated subunit where the N-terminal 146 residues of the ligand binding domain of the α7 receptor have been replaced by 27 residues from FAM7 protein [9,10,11]; the remaining sequences of α7 and Dupα7 are identical [12,13]. Dupα7 is associated with neuropsychiatric disorders, including Alzheimer’s disease [1,14], schizophrenia [15], and immunomodulation [4,16], most likely through regulating the function of the α7 receptor by direct interactions [13]. Kunii and coworkers have observed that *CHRFAM7A* was upregulated in the brains of patients with schizophrenia and bipolar disorder, and the ratio of *CHRFAM7A/CHRNA7* increased [17]. More recently, Szigeti and coworkers reported functional readouts for the *CHRFAM7A* alleles for two phenotypic readouts in mild-to-moderate Alzheimer’s disease cohort and showed 3:1 split in the population for *CHRFAM7A* carriers to non-carriers of the functional direct allele [14]. To demonstrate the translational gap, the group performed two double blind pharmacogenetic studies for both first exposure and disease modifying effect, and concluded that Dupα7 accounts for the the translational gap in development of new drugs tacking Alzheimer’s disease, and that design of future trials needs to incorporate *CHRFAM7A* pharmacogenetics [14].

It is postulated that Dupα7 acts as a dominant-negative inhibitor of α7 function, suggesting its role in human cognition and immune responses by perturbing normal α7 activities [18]. However, the exact mechanism and specific contribution of Dupα7 to the biology of α7 receptors remain highly elusive.

Even though the quaternary arrangements of both nicotinic receptors are known [3], and reports on overall stoichiometries of heteromeric α7 and dupα7 receptors have been published recently [13], atomistic details controlling those assemblies are still missing. Unravelling the molecular mechanisms governing the formation of the most probable dupα7/α7 pentamers is of high clinical interest, crucial for structure-guided approaches to target those heteromeric receptors, which account for the translational gap in research focusing on nicotinic receptors as therapeutic targets for neurodegenerative diseases [14].

To describe the most likely α7/dupα7 stoichiometry at an atomistic level of detail and to elucidate the structural, energetic and functional effects of incorporation of dupα7 monomers into the α7 channels, we combined molecular modelling, multiscale molecular dynamics (MD) simulations (all-atom and coarse-grain), and umbrella sampling (US) simulations of the whole receptors embedded in a DPPC membrane. We have also studied extracellular domains separately using all-atom MD simulations, focusing on their structure and intrinsic dynamics alongside the binding side dynamical behaviour. We have subsequently studied how the formation of α7/dupα7 heteromeric receptors affected the biding of two known ligands of α7 nicotinic receptors: the b-amyloid (Aβ_42_) and α-bungarotoxin (α-BTX) using molecular docking methods. Our results explain several experimental observations previously published. Our data link the structure and dynamics to the function of human-specific dupα7 receptors and address the specific translational gap reported for cholinergic strategies in Alzheimer’s disease [14]. The outcomes of our study may serve as the starting point for structure-guided development of potent and selective dupα7 modulators to be rendered as future therapeutics for neurological and immune disorders.

## 2. Results

### 2.1. Stoichiometry Studies for Different Combinations of Dupα7/α7

**Figure 1:**
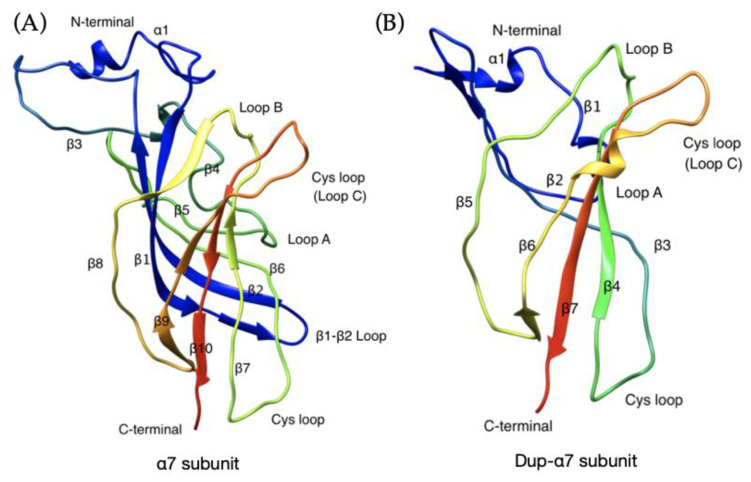
The extracellular EC domain conformation of α7 subunit (residues 1–180). (**A**) and Dupα7 subunit (**B**). The structures are coloured by gradient, from blue (N-terminus) to red (C-terminus).

**Figure 2:**
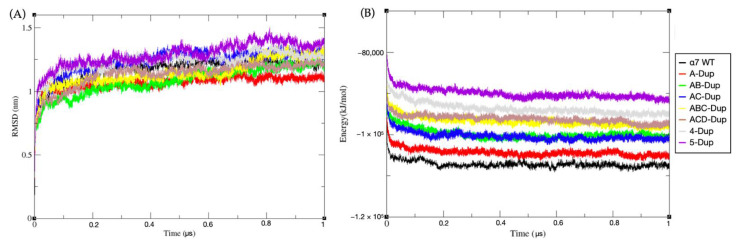
(**A**) Root-mean-square deviation (RMSD) results of eight complete transmembrane models during 1 μs CG MD simulation. (**B**) Total potential energy vs. time results of eight complete transmembrane models during 1 μs MD simulation. The black line shows the data for α7 WT model, red-A-Dup, green-AB-Dup, blue-AC-Dup, yellow-ABC-Dup, brown-ACD-Dup, grey-4-Dup, and purple-5-Dup. The schematic arrangements of all models are shown in the methods section.

The three-dimensional molecular models of extracellular domains of the canonical α7 and dupα7 are shown in Figure 1. In dupα7, the N-terminal segment (blue region in Figure 1) of α7 has been replaced by a shorter α-helix (Figure 2B). Three β-sheet segments (β1–β3 in Figure 1) are missing, yet the following segment (β-sheets 4 to 10) resembles the canonical structure.

Given that the ‘pentamer’s overall structure containing Dupα7 subunits has not been experimentally solved, we built and investigated all possible models. These different combined pentamers totalise eight models, with seven containing various combinations of Dupα7 nicotinic receptors (Figure 1B). The dimerisation assembles the functional pentamer through only two interfaces. Given this, four possible dimer combinations can be formed: WT-WT, dupα7-WT, WT-dupα7, dupα7-dupα7). Those combinations are functionally relevant because the orthosteric binding site is located at the interface between two monomers. Thus, each particular dimer combination will affect the ‘receptor’s function.

The eight models were first simulated using a coarse-grain approach by translating the atomistic model to MARTINI beads (Supplementary Figure S1). The RMSD and the total potential energy in the function of time are shown in Figure 2. All eight pentamers showed a similar magnitude of deviation from their starting structures (Figure 2A), with the average values fluctuating between 1 to 1.4 nm, which was achieved by most of the models after 50 ns of simulation.

The RMSD plots did not show any statistical difference regarding relative motions regarding their starting configuration when a single WT α7 is swapped for a dupα7. However, there is a significant difference in total internal potential energy between the different stoichiometries. As expected, the WT α7 has the lowest internal energy average value, closely followed by A-Dupα7. Interestingly, both models with two Dupα7 (AB-Dupα7 and AC-Dupα7) have undistinguishable average values, which is also the case for both models with three Dupα7 (ABC. and ACD-Dupα7). This characteristic shows that CG models could discern the different ‘stoichiometries’ internal energy, but it does not have the resolution to discern which internal configuration is more stable. Therefore, we decided to simulate all eight models with an atomistic resolution.

**Figure 3:**
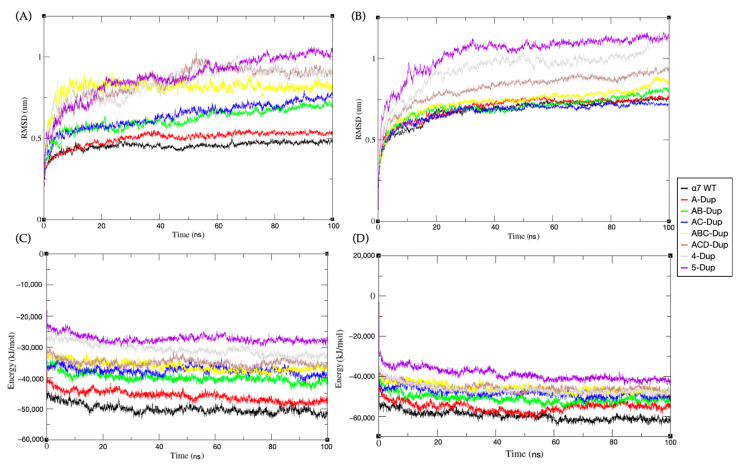
(**A**) Root-mean-square deviation (RMSD) results of eight EC domain models during 100 ns MD simulation. (**B**) Root-mean-square deviation (RMSD) of eight full-length receptor models during 100 ns MD simulation. (**C**) Total potential energy vs. time of eight EC domain during 100 ns MD simulation. (**D**) Total potential energy vs. time of eight complete transmembrane structure during 100 ns MD simulation. The canonical a7 (WT) model is shown in black, A-Dupα7-red, AB-Dupα7-green, AC-Dupα7-green, ABC-Dupα7-yellow, ACD-Dupα7-brown, 4-Dupα7-grey, and 5-Dupα7-purple. The schematic arrangements of all models are shown in methods section.

To unravel the most probable stoichiometry and the effect the interfacial interactions have on the system stability, all eight models of different combinations of Dupα7/α7 were simulated for 100 ns (each replica, all simulations performed in triplicates) in an atomistic resolution. This step was made for both the whole model embedded in a DPPC membrane and its EC domain separately. The protein RMSD for the trajectories shows that for a higher ratio of Dupα7/α7, the overall average RMSD is higher compared to the starting structure. This characteristic is more pronounced for the EC domain simulations, as shown in Figure 3A,B, respectively.

The pentamer simulations embedded in membrane show RMSDs quickly plateauing for most of the models containing less than three Dupα7 subunits. Nonetheless, the ACD-Dupα7 model shows a higher average RMSD than its ABC-Dupα7 counterpart, which may indicate that interfacial dupα7-dupα7 interactions may be favourable for the molecular assemble.

The analysis of the pentamers’ internal potential energy (Figure 3C,D) shows that the average energy increases as the Dupα7/α7 ratio increases. For the extracellular (EC) domain, the combination containing a single Dupα7 subunit has significantly lower energy than all the other models. However, the full-length receptor model simulations show that the A model’s internal energy (Figure 3B) fluctuates between similar values to the WT and AB model. The difference in energy between the EC domain and its counterpart containing all three domains may indicate that the transmembrane and intracellular (IC) domains may significantly stabilise the pentamers containing Dupα7 subunits.

**Figure 4:**
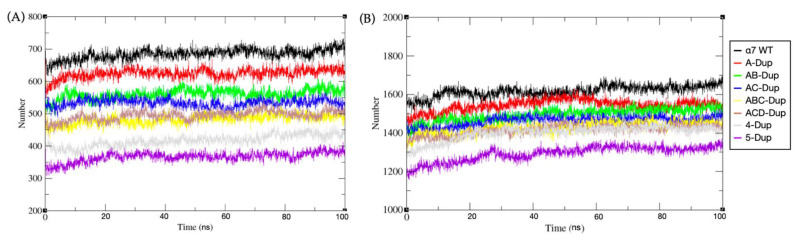
(**A**) The total number of hydrogen bonds vs. time of the eight EC domain combinations. (**B**) The total number of hydrogen bonds vs. time of eight fully models during 100 ns MD simulation. The black line shows α7 WT model; red-A-Dupα7, green-AB-Dupα7, blue-AC-Dupα7, yellow-ABC-Dupα7, brown-ACD-Dupα7, grey-4-Dupα7, and purple-5-Dupα7. The schematic arrangements of all models are shown in the methods section.

As expected, the total number of hydrogen bonds formed shows similar behaviour to the calculated potential energy. Specifically, the EC domain trajectories (Figure 4A) show a higher number of H-Bonds for both WT and A-Dup than their counterparts. The pentamers containing a higher number of Dupα7 subunits show higher average internal energy and a lower average number of hydrogen bonds. Therefore, a clear correlation was observed between a higher Dupα7/α7 ratio and lower structural energy and fewer total hydrogen bonds (Figure 4B).

When observing the hydrogen bond formation between interfaces, several keys residues were identified. All these sets of residues are listed in Supplementary Tables S1–S16. Investigating WT-WT dimer, we identified six residues that are often present on the interaction between two adjacent subunits (N69, N75, R101, P110, D111 and W172). In contrast to that, the number of interfacial hydrogen bond pairs for WT-Dupα7 interfaces varied between three to four. One of the main differences between the WT-WT interfaces and Dupα7 containing interfaces is that the latter does not have the hydrogen bond between R101 and the P110 of the sequential subunit residues are not present in the sequence of the Dupα7.

**Figure 5:**
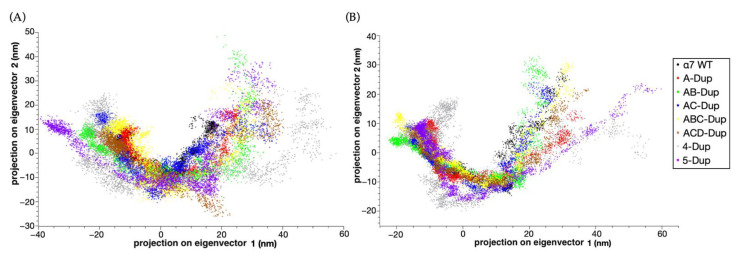
(**A**) Principal component analysis, showing the 2D projection of eight different models of the extracellular (EC) domain. (**B**) Principal component 2D projection of eight different fully models during 100 ns of the atomistic MD simulation. The data for α7 WT model is coloured black; A-Dupα7-red, AB-Dupα7-green, AC-Dupα-blue, ABC-Dupα7-yellow, ACD-Dupα7-brown, 4-Dupα7-grey, 5-Dupα7-purple. The schematic arrangements of all models are shown in the methods section.

A higher number of Dupα7 subunits directly changes the molecular dynamics. Figure 5 shows that the trajectory projection on the two most significant principal components is affected by the dupα7/α7 ratio. WT, A-Dupα7 and the complexes with two Dupα7 (AB and AC) subunits have similar distributions (Figure 5A), especially for the transmembrane complexes (Figure 5B). The most significant difference comes from models with three or higher number of dupα7 subunits. Given their higher energy, pentamers with four or five subunits resulted in higher magnitudes on their projection values, achieving a sparser distribution on the two-dimensional principal component space. These complexes show the same behaviour for their extracellular (EC) domain simulations, albeit less pronounced than the complexes containing a lower Dupα7/α7 ratio when the EC domain is simulated by itself.

**Figure 6:**
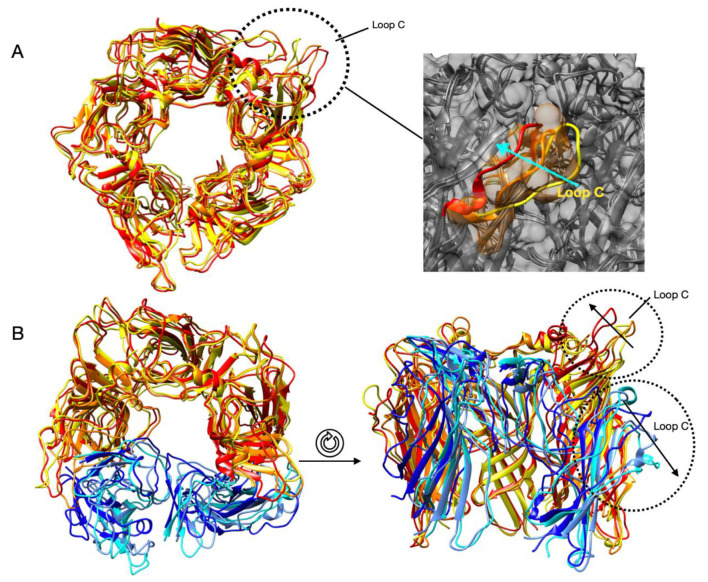
(**A**) Left panel: The extracellular view of the three conformations of the α7 model EC domain. Right panel: the Loop C motion. The representative configurations sampled around 15 ns, 50 ns and 85 ns are coloured yellow, orange and red, respectively. (**B**) The extracellular view of the three conformations of the AB-Dupα7 model EC domain. The representative configurations for the starting conformation’s state (around 15 ns) are coloured yellow and cyan; the representative configurations for the state sampled around 50 ns are coloured orange and cornflower blue; the representative configurations for state sampled around 85 ns are coloured red and blue. The dashed lines represent the interfacial loops, which are the areas with the highest fluctuation; the arrows within the dashed circles represent the low-amplitude motions within the loop C for both WT and Dupα7.

These differences in the transmembrane model dynamics are directly related to the motions on its EC domain, mainly of loop C and the loops connecting the central β sheets (Figure 6). The motion of Loop C changes depending on its neighbouring subunit for both simulated models, as shown in Figure 6 and Supplementary Figure S2. For WT-WT interfaces (Figure 6A), the loop fluctuates between an open and closed conformation and the entire α7 model remains stable overall (Supplementary Figure S3). WT-Dupα7 and Dupα7-Dupα7 interfaces (Figure 6B) show a more open conformation and higher local flexibility.

### 2.2. The Effect of Dupα7 Subunits on Ca^2+^ Conductance

**Figure 7:**
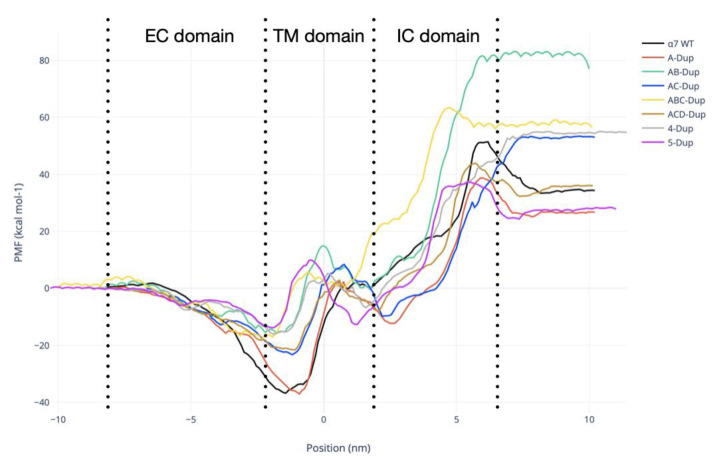
Potential of mean force (PMF) calculated for the position of the Ca^2+^ moving through the pentamer axis. The black line shows the PMF obtained for the canonical α7 (WT) receptor, A-Dupα7-red, AB-Dupα7-green, AC-Dupα7-blue, ABC-Dupα7-yellow, ACD-Dupα7-brown, 4-Dupα7-grey, and 5-Dupα7-purple. The schematic arrangements of all models are shown in Figure 8B.

Umbrella sampling simulations were carried out to investigate the effect of different combinations of Dupα7/α7 subunits on the Ca^2+^ intake. As shown in Figure 7, all eight potential mean force (PMF) curves show similar profiles. At −8 nm, the ion is at the tunnel entrance in the EC domain, transitioning to the transmembrane region (TM) around −1.5 nm. The energetic differences between the eight combinations started to arise around −5 nm, near the TM ‘region’s start. At this region, α7 and A-Dupα7 have the lowest energy (−37.5 kcal.mol^−1^). However, an energetic barrier emerges after the TM entrance, which is higher for AB, ABC, 4-Dupα7 and 5-Dupα7. This energetic difference emerges from how the ions interact with the model, specifically with glutamate E254. The canonical α7 (WT) structure can maintain the beginning of the α-helix located in the TM entrance, where the E254 residue is located (Sequence: 250-LVAEIM-257). This behaviour allows a more favourable interaction with the E254, resulting in a higher number of contact points with Ca^2+^ (Supplementary Figure S4 and Supplementary Tables S17 and S18).

The final 2–6 nm section represent the intracellular (IC) domain, which shows that the energy becomes significantly higher when the ion far away from the TM domain. The AB-Dupα7 shows the highest energy (81.2 kcal.mol^−1^) at the bottom of the IC domain, with the lowest 5-Dupα7 model results (28.3 kcal.mol^−1^) as shown in Table 1

### 2.3. The Effect of Dupα7 Subunits on Macromolecular Ligand Binding

Alongside the effect of the Dupα7/α7 ratio on the pentameric receptor dynamics, we also assessed the effects of Dupα7/α7 ratio on the binding of two macromolecular ligands: α-bungarotoxin (α-BTX) and the amyloid β (Aβ_42_). α-BTX is a well-established, 74 residues (8 kDa) neurotoxin that binds nicotinic acetylcholine receptors, including a7 subtypes, and acts as a competitive antagonist upon them. While the experimental structure of α-BTX-α7 is known (PDB code: 4HQP), the structural information on interactions between receptors containing Dupα7 subunits and α-BTX is missing. Aβ_42_ and α7 interact with high affinity [19,20]. However, the details of those interactions remain elusive.

To gain insight into those interactions, we have performed molecular docking calculations. Molecular models of the α-BTX binding sites were built on the dimerisation interface (orthosteric binding sites, inferred from the reported α-BTX-α7 interactions). We evaluated the binding poses of α-BTX and their binding affinities to all four combinations of receptors: α7/α7 (canonical), α7-Dupα7, Dupα7-α7, and Dupα7-Dupα7.

To identify the binding site for Aβ_42,_ we performed the exhaustive scan of the whole extracellular receptor domain using ClusPro. The best-scoring poses (8/10) converged to the site, which partially overlaps with the reported α-BTX binding sites, consistent with the reports on α7 receptor activation via orthosteric modality reversing the Aβ_42_ binding [20]. Therefore, we concluded that the amyloid β (Aβ42) binding site overlaps with that for α-BTX.

The results are shown in Table 2. α-BTX showed very low binding affinity to all the receptors containing Dupα7 subunits (Table 2), suggesting that those receptors will be resistant to α-BTX. This observation agrees with the published experimental data showing that CHRFAM7A decreased α-BTX binding as detected by immunohistochemistry and flow cytometry and markedly decreased α-BTX staining neuromuscular junction of CHRFAM7A transgenic mice [21].

For Aβ_42_ interactions, the ligand residues that interacted with the receptor binding sites were located in the C-terminal regions of the Aβ_42_ monomer (Table 2). The predicted binding affinity of the Aβ_42_ to the binding site comprising two α7 subunits (the canonical receptor) was in the low nM range, consistent with available experimental data [20]. As a comparison, α-BTX was predicted to bind to the canonical a7 binding sites with one order of magnitude lower (high pM range) than Aβ. This difference, too, is consistent with the experimental data available.

**Figure 8:**
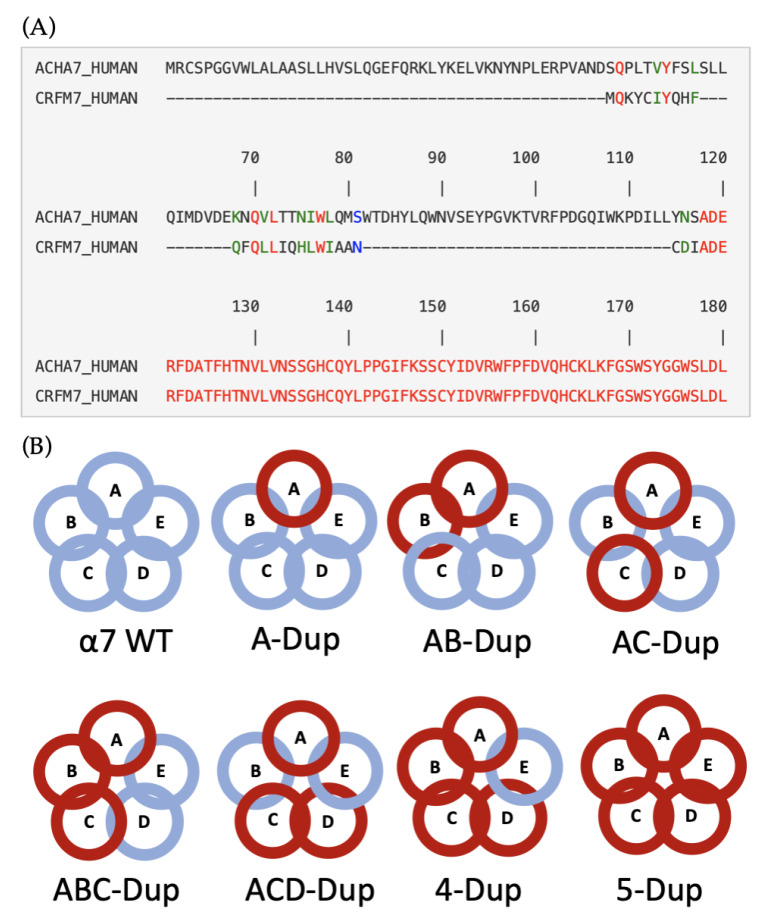
(**A**) Sequence alignment of α7/dupα7 extracellular (EC) domains (residues 1–180), performed by ClustalW, green are the residues with high similarity and in red the conserved residues. (**B**) Schematic representation of all eight different model arrangements dupα7-α7 pentamer, considered in this study: the canonical (WT) α7 subunits are coloured blue; dupα7 subunits are coloured red.

The α7 receptor residues crucial for maintaining the binding pose include R208, F209, and E211. All those residues are conserved in both α7 and Dupα7 isoforms, albeit their dynamics are different in Dupα7. In particular, R208 and E211 were involved in favourable, stabilising electrostatic interactions with the ligand. F209 is involved in the network of aromatic residues, including two tryptophan residues: W171 and W77. The latter is located at the different subunit, as showed in Figure 8. Despite variation in the N-terminal region of the receptor, this residue is conserved in primary sequence alignment between α7 and Dupα7 (Figure 8A).

When we analysed the effects of Dupα7 on the binding affinities, we observed a more favourable predicted binding affinities for Aβ by both α7-Dupα7 and Dupα7-Dupα7 (predicted to be within low-mM range), compared to the canonical α7-α7 binding site (affinities in low-nM range, Table 2). Intriguingly, calculated binding affinity for Aβ to Dupα7-α7 improved (high nM to low μM range). This difference indicates that the residues involved in the interactions with Aβ and are not conserved between Dupα7 and α7 (S56, S58, L60, and Q61) may be necessary complex stabilisation. Additionally, it indicates that the α7 secondary structure architecture in this region is critical for the binding of Aβ_42_. This characteristic suggests that receptors containing Dupα7 subunits will be more resistant to Aβ_42_-related toxicity, supported by recent experimental data [14].

## 3. Discussion

### 3.1. Dupα7/A7 Ratio Directly Affects the Structural Cohesion of the Receptor Pentamer

In this work, we aimed to understand the assembly and stability of different combinations of functional α7 nicotinic acetylcholine receptors (nAChR), bearing a partial duplicate Dupα7nAChR. Lasala and coworkers reported that WT α7 nicotinic receptors could form functional pentamers incorporating Dupα7 subunits. However, the minimum number of WT α7 had to be two [13]. Nonetheless, neither the effect of different stoichiometry on α7 nAChR receptor function nor the structural stability of pentamers containing Dupα7 subunits is known.

Our results showed that the overall arrangement of the extracellular domain for both α7 and Dupα7 subunits is similar, except for the N-terminal portion of the EC domain. This characteristic agrees with the models published shown by Lasala and coworkers [13]. Their models showed that changes in the α1 loop and differences in the configuration of the β-sheets in the EC domain core could be observed between the canonical and receptors with Dupα7 subunits. Our data confirmed that these modifications affected the ‘heteropentamers’ stability and showed and how the interfaces interact.

We showed how structural equilibration of the receptors with different stoichiometries occurs through time. For the pentamers with higher Dupα7 content, the RMSD curves take longer to plateau, and they reach higher average values than their starting structure, which reflects their decreased stability and weaker cohesion. These characteristics are found in both resolutions used: coarse-grain and atomistic MD as well. Additionally, similar behaviour when comparing the internal structural energy can be found: the ‘pentamers’ average structural energy steadily increases with the Dupα7/α7 ratio. This increase comes from several hydrogen ‘bonds’ dissemble and favourable interactions, both at the interfaces and hydrophobic core. One of the critical differences comes from the absence of the interaction with the R101 in the receptors containing Dupα7 subunits. This residue interacts with several other residues located in the subsequential subunit, and the lack of these interactions directly affects the structural cohesion of the interface.

The truncation directly affects the orthosteric binding site configuration. As we showed, the loop dynamics that works as a gatekeeper entirely depends on the Dupα7/α7 interactions.

The canonical a7 (WT) pentamer simulations showed that loop C moves from an open conformation to a closed conformation. At the WT-Dupα7 interfaces, very different dynamic signatures are found. As discussed in previous works [5,22], the requirement for a functional α7 pentamer is a single functional orthosteric binding site, albeit this is less sensitive in comparison to a fully functional pentamer with up to five orthosteric sites. Given the destabilisation effect that dupα7 subunit causes on its dimerisation interface and in the orthosteric binding site, the dupα7 interface should be less sensitive to the orthosteric ligand binding. This effect is evidenced by how the loop C on the dupα7 subunit moves away from the pentamer. This movement shows that the dupα7 binding site might remain open, not stabilising the orthosteric ligand in the binding cavity.

The partial duplication and truncation that leads to the dupα7 protein are located in the extracellular domain (Figure 8A). Hence, this is the region that shows the highest difference between WT and dupα7 dynamics. However, the transmembrane domain reduces the ‘system’s overall energy by creating a higher number of hydrogen bonds, resulting in a lower average RMSD and RMSD standard deviation compared to the EC domain by itself. Nonetheless, pentamers containing Dupα7 subunits go through structural changes within the TM domain, mainly on the entrance region (residues 250–257). The energetic landscape referring to the extracellular to intracellular calcium intake showed that this region plays a vital role in this process.

Residues 250–257 go from an α-helical conformation in the WT pentamer to a short π-helix in most dupα7 models. This conformation transition destabilises the favourable interactions between the calcium and the residue E254 and its neighbouring residues, which was sampled in the WT simulation. This transition also is an effect that is directly related by the Dupα7/α7 ratio: Both WT and A-Dupα7 models show similar profiles, indicating that a single Dupα7 subunit does not affect the calcium intake substantially. Nonetheless, another Dupα7 subunit increases the system energy substantially for the TM domain entrance region. This energy difference suggests that pentamers bearing two consecutive Dupα7 subunits are unlikely to occur.

These effects are also position-dependent: AB-dupα7 model and ACD-dupα7 model showed similar energetic profiles within the calcium intake TM. These profiles had lower free energy values than the AC-dup α7 model in the TM region. This indicates that Dupα7 interfaces have a less pronounced effect on the function of the Dupα7-α7 interface for models with more than one Dupα7 subunit. The 250–257 loop interactions and the calcium are significantly reduced for models with four or five subunits. A higher barrier is found in comparison to the other models.

Hence, this shows that pentamers with four or five dupα7 subunits are not functionally viable. In addition to the previously published works, the results shown in this work shed light on the plausible stoichiometry of Dupα7/α7 subunits. We evaluated stability and functional differences emerging from the positioning of the Dupα7 subunits within the pentamer. Albeit with a weaker interface interaction, the AC-dupα7 model showed a lower effect on the calcium transition than the AB-dupα7 model. A similar effect was observed when the pentamer had three dupα7 subunits. The simulations of models with four or five Dupα7 are both too unstable, with an unfavourable effect on the Ach binding site organisation compared to the crystal structure. Additionally, it disrupts the calcium transmission by the pentamer.

In summary, a number higher than three Dupα7 subunits is unlikely to be naturally occurring and to be functional. The most stable stoichiometry is the 1: Dupα7-4:WT α7, which is also one of the combinations with the least negative effect on calcium transmission. With two subunits, we expect to see Dupα7 interacting with WT subunits, given it has a lower effect on its function. Naturally occurring pentamers with three subunits should be less likely than with two, but should also be more favourable the interaction between Dupα7/α7 interfaces, which we would expect the predominance of an ACD dupα7 organisation.

### 3.2. Dupα7/A7 Ratio Affects the Ligand Binding and May Be Linked to the Nicotinic Translational Gap

The α7 receptor has been a promising target for diseases affecting cognition. However, the results gathered in animal studies failed to translate into human clinical trials identifying a translational gap. As CHRFAM7A is human-specific, it was not included in those preclinical studies, and effects arising from its distinct structural and dynamic features were not taken into account. As the CHRFAM7A gene is present in different copy number variations in the human genome with high frequency [19], understanding distinct features of dupα7 may offer novel insights when exploring the human α7 receptor as a drug target.

Recent reports have shown the direct interactions between α7 receptors and Aβ_42_ [23,24,25]. These studies strongly suggest that the α7 receptor can contribute to synaptic dysfunction in ‘Alzheimer’s disease (AD) as Aβ oligomers can alter neuronal signalling through interactions with nicotinic receptors, particularly with α7. However, how exactly Aβ interacts with α7 receptor, and whether human-specific dupα7 increases or decreases those interactions, has not been fully understood.

Regarding interactions with disease-linked macromolecular ligands, our results indicate that in mixed functional receptors (i.e., comprising of both a7 and dupα7 subunits), dupα7/α7 interfaces can bind Aβ_42_ with a higher affinity than α7/dupα7 and dupα7/dupα7, albeit impaired compared to canonical α7/α7 sites. Receptors bearing Dupα7 subunits are shown to be insensitive to α-BTX. These results collectively suggest that the receptors bearing Dupα7 subunits may be less sensitive to effects exerted by neurotoxin or Aβ_42_. This data is in agreement with the recent study, which focused on the function of *CHRFAM7A* alleles in vitro in two disease-relevant phenotypic readouts: electrophysiology and Aβ uptake, and in the double-blind pharmacogenetic analysis on the effect of therapy using acetylcholine esterase inhibitors (AChEIs), based on *CHRFAM7A* carrier status [14].

Mechanistic insights arising from our work suggest competitive binding between α-BTX (an orthosteric ligand) and Aβ_1–42_ to the α7 receptors. The earlier studies support this characteristic, showing that both orthosteric agonists and antagonists mitigate Aβ uptake [14]. Uptake of Aβ_1–42_ via α7 receptors binding induces apoptosis, and orthosteric α7 agonists mitigate the Aβ-induced apoptosis in animal models as reported by Szigeti et al. and references therein [14]. The results of our study highlight the mechanistic link between receptor structure and Aβ binding, indicating key differences between α7 receptors and receptors bearing dupα7 subunits, which may be translated to the clinic. Moreover, our results suggest that in receptors containing dupα7 subunits, Aβ_42_ might be competitive to α-BTX, albeit its binding affinity is low, hence the significance of this potential competing is challenging to estimate. Further follow-up studies are needed to validate these findings.

At the time of translation to the clinical trials, virtually all drugs effective in animals have demonstrated a lack of efficacy in humans, showing a robust translational gap. Dupα7 functional studies are sparse and are lacking in the clinical context. Clinical efforts need to be continued with a trial design incorporating Dupα7 distinct structural biology, pharmacology and pharmacogenetics. Dupα7 non-carriers account for 25% of the ‘Alzheimer’s disease (AD) population, which is significant, considering an increasing number of AD patients. Our results, which match neuronal toxicity data published [14], suggest that Dupα7 carriers should be protected against Aβ effects to some extent, and Dupα7 non-carriers should be more acutely affected by Aβ effects. Therefore, therapeutics that reduce amyloid burden could be effective in non-carriers. Considering the number of AD patients worldwide and AD being essentially an unmet clinical need, these findings pave the way to bring new AD therapeutics into the clinic.

## 4. Materials and Methods

### 4.1. Molecular Modelling of α7 and Dupα7 Receptors

The initial models of pentameric α7 homopentamers and partially duplicated dupα7 subunits were created using SWISS-MODEL, a fully automated protein structure homology-modelling server, accessible via the ExPASy web server [26,27]. The primary sequences of the human canonical α7 and dupα7 were obtained from the UniProt repository (entries P36544 and Q494W8, respectively). Fifty models were generated and ranked according to their sequence similarity and QMEAN [26] quality scores combined. After a visual inspection procedure for the top 10 ranked molecules, the model based on the crystal structure of α7-AChBP in complex with lobeline (PDB code: 5AFH) combined with high-resolution (4.3 Å) cryo-EM structure of mouse 5-HT_3_ serotonin receptor (PDB code: 6BE1) was chosen for both receptors as the difference comes mainly from the N-terminal region (Figure 8A). Different stoichiometries were generated in the UCSF Chimera molecular modelling and visualisation toolkit [28] by overlayin the α7 WT subunit with dupα7 in eight receptor models in total (Figure 8). A disulfide bridge at the conserved Cys-loop was ensured for each model. All models, arising from different stoichiometries of dupα7 and α7 subunits, were quality checked by UCSF Chimera, having any missing loops modelled by MODELLER interface [29,30] within UCSF Chimera, and conformations of interfacial side chains checked for steric clashes.

### 4.2. Coarse-Grain Molecular Dynamics Simulations

To equilibrate the modelled pentamers and understand the intrinsic dynamics in a time scale relevant to the ion channel conductance, coarse-grain simulations of all eight models were done. The atomistic models were translated to MARTINI beads [31] and parametrised with the MARTINI 2.2 force field [32]. The principal protein axis was aligned to the Cartesian Z-axis. The membrane was built using the insane.py script. The membrane model was built using dipalmitoylphosphatidylcholine (DPPC), with 15 nm in the X and Y axis and 25 nm in the Z-axis, resulting in a box of 15 × 15 × 25 nm in dimensions. The system was then solvated, using 90% of MARTINI water beads and 10% anti-freeze MARTINI water beads [32]. The solvated receptor-membrane systems were energy minimised using steepest descent algorithm and equilibrated. In the minimisation, the energy step size was set to 0.001 nm, and the maximum number of steps was set to 50,000. The minimisation was stopped when the maximum force fell below 1000 kJ/mol/nm using the Verlet cutoff scheme. Treatment of long-range electrostatic interactions and Van der Waals interactions were set to be shifted to 0 and 0.9 nm, respectively, beyond the cutoff of 1.5 nm. After the energy minimisation, heating to 300 K was performed for 10 ns with a time step of 20 fs. The temperature coupling was set between the protein and the non-protein entities using a Berendsen thermostat, with a time constant of 1 ps and the temperature set to reach 300 K with the pressure coupling. Pressure equilibration was run at 300 K with a semi-isotropic Berendsen barostat and set to 1 bar in an NPT ensemble. Both NVT and NPT had harmonic position restraints were applied to the backbone. The constraint algorithm used was LINCS. The production run was made using the same parameters as NPT, except the backbone position restraints were removed. The production run was made in triplicates of 1 μs each, for each of the eight combinations of α7/dupα7.

### 4.3. Atomistic Molecular Dynamics (MD) Simulations

Atomistic MD simulations have been carried out to generate ensembles to get a detailed insight into the stoichiometry of nicotinic α7/dupα7 receptors in atomistic resolution. The simulations were performed for α7/dupα7 pentamers with different stoichiometries for the models containing all three domains (EC-TM-IC) and only the EC domain to evaluate the effect of the TM-IC domain on the dynamics.

All simulations were performed using Gromacs 2016.3 [33]. The protein was parametrised using the AMBER99SB-ILDN force field, with the DPPC lipid bilayer and TIP3P water model [34]. The α7 and dupα7 models were embedded in a DPPC bilayer lipid molecule, using the computational membrane builder tool in the CHARMM-GUI server (www.charmm-gui.org, accessed on 24 April 2021) [35,36,37]. Box distance was set to 1 nm, and periodic boundary conditions were applied. The box was solvated and Na^+^ and Cl^−^ ions were added to achieve a 0.1 M concentration and maintain charge neutrality of the unit. The solvated receptor-membrane systems were energy minimised and equilibrated. The minimisation ran using steepest descent for 1000 cycles followed by the conjugate gradient. Energy step size was set to 0.001 nm, and the maximum number of steps was set to 50,000. The minimisation was stopped when the maximum force fell below 1000 kJ/mol/nm using the Verlet cutoff scheme. Treatment of long-range electrostatic interactions was set to Particle Mesh-Ewald (PME) [38], and the short-range electrostatic and van der Waals cutoff set to 1.0 nm. After the energy minimisation, heating to 300 K was performed for 20 ps with a time step of 2 fs and position restraints applied to the backbone in an NVT ensemble. The constraint algorithm used was LINCS, which was applied to all bonds and angles in the protein [39]. The cutoff for non-bonded short-range interaction was set to 1.0 nm with the Verlet cutoff scheme. Long-range electrostatics were set to PME. The temperature coupling was set between the protein and the non-protein entities using a Berendsen thermostat, with a time constant of 0.1 ps and the temperature set to reach 300 K with the pressure coupling off. Pressure equilibration was run at 300 K with a Parrinello–Rahman barostat and set to 1 bar [40] in an NPT ensemble. The equilibration trajectories were set to 5 ns (discarded from the analysis), and the production MD simulations were performed for 100 ns. Each trajectory was run in triplicates.

Analysis of the trajectories was performed using GROMACS tools, including root-mean-square deviation (RMSD) to assess overall stability, per-residue root-mean-square fluctuation (RMSF) to assess the local flexibility, and calculating SASA for solvent-mapping.

### 4.4. Umbrella Sampling (US) Simulations

Umbrella sampling (steered molecular dynamics) [41] simulations were used to assess the influence of different stoichiometry of receptors on the ‘pentamer’s ion conductance. The energies of the Ca^2+^ ion pulled through the axis of the channel pore (Z-axis) were calculated using the Weighted Histogram Analysis Method (WHAM) method [42] to extract the potential of mean force (PMF). To prevent the channel from moving out of the membrane, the receptor subunits were position-restrained during the pulling simulations, using 1000 kJ mol^−1^nm^−2^. The ion has been placed above the top of the EC domain and pulled downward along the Z-axis towards the TM and IC domains over 5 ns at a rate of 0.01 Å/ps. A series of umbrella sampling windows were generated from the pulling trajectory to proceed with the umbrella sampling. The entire pathway covering the range of [−10, 10] Å was divided into 0.7 Å, totalizing 40 windows.

### 4.5. Aβ42 and α-Bungarotoxin (α-BTX) Binding to α7 Pentamers

The analysis of interactions between different α7/dupα7 receptors and two established macromolecular ligands: Aβ and α-BTX, were performed by molecular docking. Aβ (PDB code: 6RHY) and α-BTX (PDB code:4HQP) were docked to all four possible combinations of α7 and dupα7 interfaces using ClusPro web server [43,44]. The top 10 lowest-energy complexes were selected to further analysis, and the binding affinities were calculated by SeeSAR (www.biosolveit.de, accessed on 24 April 2021) [45], using the HYDE scoring function [46].

## 5. Conclusions

In this work, we performed a systematic study on all possible combinations of Dupα7/α7 nicotinic receptors, focusing on their structural stability and stoichiometry, to find the most probable functional pentamers bearing Dupα7 subunits. This understanding was essential since Dupα7 has been regarded as a dominant-negative regulator of α7 receptors. However, reports of functional pentamers bearing Dupα7 subunits have been published. To address the conflicting evidence from published studies, we modelled all possible combinations and evaluated them using structure-based multiscale computational methods. We showed that higher content of Dupα7 subunits resulted in less cohesive pentamers, and dupα7/dupα7 interfaces, corresponding to the orthosteric binding sites, were markedly less stable than Dupα7/α7 interfaces. These indicate that the most likely combinations were pentamers bearing one Dupα7 subunit (A-dupα7 model) or pentamers containing two non-consecutive subunits (AC-dupα7 model). Pentamers bearing three subunits with the lowest dupα7/dupα7 interfaces (ACD-dupα7 model) were also suggested to be functional via analysis of the energetic landscape carried out via umbrella sampling simulations. The comparative studies of the energetic landscapes for the pentamers with different stoichiometries showed that receptors with low ratio of Dupα7/α7 are still functional, even though higher energy barriers are observed for these pentamers. On the other hand, the increase of the number of Dupα7 subunits negatively affected the Ca^2+^ uptake via the receptor. Our work has also shown that dupα7 interfaces are insensitive to α-bungarotoxin (α-BTX), but not to Aβ_42_, even though their Aβ_42_ binding is impaired compared to the canonical α7 receptors. This impairment indicates that receptors containing dupα7 subunits are less sensitive to Aβ_42_ effects and that dupα7 subunits, despite their impaired agonist binding, may offer protection against detrimental Aβ_42_ effects. We expect that our work will contribute to the elucidation of the biological roles of Dupα7 subunits, generating models that can be used for a rational drug design. Future research aiming to characterise the function of Dupα7 in the clinical context may result in novel pathways for AD treatment based on early-stage preclinical data. As α7 receptors are implicated in a broad range of diseases, including cognition, memory, schizophrenia, chronic pain and inflammageing, mechanistic insights into receptors containing Dupα7 subunits will impact these therapeutic areas, including those conditions which currently represent an unmet clinical need.

## Figures and Tables

**Table 1:** Sampling results Ca^2+^ permeability energetics.

Energy/Model (kcal. Mol^−1^)	α7	A-Dup	AB-Dup	AC-Dup	ABC-Dup	ACD-Dup	4-Dup	5-Dup
EC -> TM (around-1.5 nm)	−37.5	−37.5	−15.7	−23.6	−16.4	−21.6	−15.9	−13.9
Transmembrane domain (around 0 nm)	0.2	1.8	14.6	7.7	4.1	2.3	4.8	9.84
TM –> IC (around 2 nm)	−0.1	−12.5	−0.1	−10.4	1.1	−7.6	−6.6	−12.6
Exiting IC (around 6 nm)	51.1	37.6	81.2	52.3	63.1	43.7	52.8	37.3

**Table 2:** Binding affinity ranges of Aβ_42_ or α-BTX to orthosteric binding sites. All affinity ranges were calculated by SeeSAR.

Binding Site Interface	Aβ_42_ Affinity (K_i_ calc)	a-BTX Affinity (K_i_ calc)
α7-α7	Low nM	High pM
α7-Dupα7	mM	>mM
Dupα7-α7	High nM to Low μM	>mM
Dupα7-Dupα7	mM	>mM

## Data Availability

Data available upon request.

## Supplementary Materials

The Supplementary Materials are available online at https://www.mdpi.com/article/10.3390/ijms22115466/s1.
