# Supplementary material for: Structure, Dynamics, and Ligand Recognition of Human-Specific CHRFAM7A (Dupα7) Nicotinic Receptor Linked to Neuropsychiatric Disorders
Source: Int J Mol Sci. 2021 May 22;22(11):5466. doi: 10.3390/ijms22115466 (PMC8196834; doi:10.3390/ijms22115466)
Supplement: Supplementary file 1 [file ijms-22-05466-s001.zip › ijms-1220608-supplementary.pdf]

Supporting Information

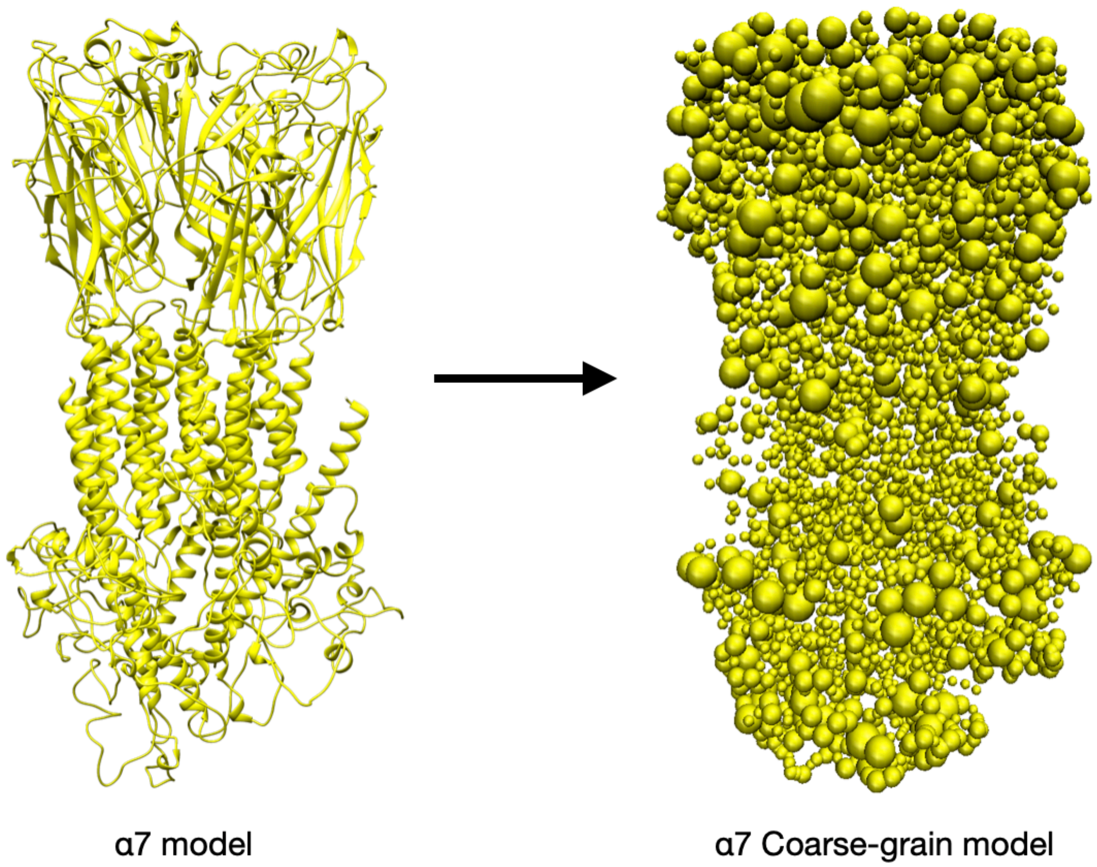

Figure S.1: The atomistic α7 model translation to MARTINI coarse-grain framework.

Table S.1: Full list of interfacial hydrogen bond contacts for the canonical α7 receptor (WT) before MD simulations.

Donor		Acceptor		Donor-Acceptor distance (Å)	Donor hydrogen-Acceptor distance (Å)	Total number of contacts
AB (WT-WT)	10 H-bonds					171
GLN	61.A NE2	SER	149.B OG	2.868	1.887	
ASN	75.A ND2	ARG	121.B O	2.983	2.166	
ARG	101.A NE	TYR	173.B OH	2.837	1.823	
ARG	101.A NH1	TYR	173.B OH	3.388	2.633	
ARG	101.A NH2	PRO	39.B O	3.345	2.583	
ARG	101.A NH2	LEU	40.B O	3.013	2.183	
ASN	69.B ND2	MET	63.A O	2.815	1.808	
ASN	69.B ND2	ASP	64.A OD1	2.834	1.909	
GLN	70.B NE2	PRO	192.A O	3.623	2.651	
TRP	171.B NE1	LEU	141.A O	2.842	1.936	
BC (WT-WT)	5 H-bonds					219
ASN	75.B ND2	GLU	120.C OE1	2.685	1.809	
ARG	101.B NH1	SER	172.C O	3.267	2.429	
ASN	133.B ND2	TYR	173.C OH	3.189	2.322	
TRP	171.C NE1	LEU	141.B O	2.777	1.77	
SER	172.C OG	ASN	133.B O	2.843	1.908	
CD (WT-WT)	5 H-bonds					164
LYS	28.C N	GLU	41.D OE2	3.458	2.628	
TRP	77.C NE1	ASP	119.D O	2.975	2.063	
GLN	70.D NE2	PRO	192.C O	3.183	2.218	
SER	149.D OG	GLN	61.C OE1	2.823	1.911	
TRP	171.D NE1	LEU	141.C O	2.797	1.795	
DE (WT -WT)	10 H-bonds					123
GLN	61.D NE2	SER	149.E OG	2.955	2.019	
TRP	77.D NE1	GLU	120.E OE1	2.748	1.864	
TRP	77.D NE1	GLU	120.E OE2	2.887	1.964	
ARG	101.D NH1	LYS	109.E O	3.12	2.329	
ARG	101.D NH2	ASP	111.E OD1	2.66	1.749	
ASN	129.D ND2	SER	172.E OG	2.876	1.855	
GLN	70.E NE2	PRO	192.D O	2.982	1.977	
SER	117.E OG	GLY	189.D O	2.819	2.229	
ARG	121.E NH1	PRO	143.D O	2.818	1.995	
TRP	171.E NE1	LEU	141.D O	2.808	1.806	
EA (WT-WT)	13 H-bonds					185
ASN	69.A ND2	MET	63.E O	2.867	1.858	
GLN	70.A NE2	PRO	192.E O	3.007	2.022	
ARG	121.A NE	ASN	75.E OD1	2.826	2.012	

ARG	121.A NH1	ASN	75.E OD1	2.855	2.005	
TRP	171.A NE1	LEU	141.E O	2.78	1.844	
SER	172.A OG	ASN	129.E OD1	2.833	1.943	
GLN	61.E NE2	SER	149.A OG	2.974	2.171	
TRP	77.E NE1	ASP	119.A O	2.684	1.953	
ARG	101.E NE	TYR	173.A OH	2.872	1.87	
ARG	101.E NH1	TYR	173.A OH	3.428	2.629	
ARG	101.E NH2	PRO	39.A O	3.361	2.439	
ARG	101.E NH2	LEU	40.A O	2.843	2.132	
ASN	129.E ND2	ASP	111.A OD2	2.675	1.806	

Table S.2: Full list of interfacial hydrogen bond contacts in A-dupα7 receptor before MD simulations.

Donor		Acceptor		Donor-Acceptor distance (Å)	Donor hydrogen-Acceptor distance (Å)	Total number of contacts
AB (dup- WT)	3 H-bonds					132
GLN	70.B NE2	PRO	102.A O	3.452	2.486	
ARG	121.B NH1	PHE	32.A O	3.467	2.54	
TRP	171.B NE1	LEU	51.A O	2.964	2.141	
BC (WT-WT)	4 H-bonds					214
ASN	75.B ND2	GLU	120.C OE1	2.714	1.989	
ARG	101.B NH1	SER	172.C O	3.492	2.74	
ASN	133.B ND2	TYR	173.C OH	3.115	2.243	
TRP	171.C NE1	LEU	141.B O	2.71	1.699	
CD (WT-WT)	3 H-bonds					158
TRP	77.C NE1	ASP	119.D O	2.909	2.017	
GLN	70.D NE2	PRO	192.C O	3.233	2.255	
TRP	171.D NE1	LEU	141.C O	2.787	1.78	
DE (WT-WT)	8 H-bonds					131
GLN	61.D NE2	SER	149.E OG	2.97	2.111	
TRP	77.D NE1	GLU	120.E OE1	2.675	1.821	
TRP	77.D NE1	GLU	120.E OE2	3.006	2.199	
ARG	101.D NH1	LYS	109.E O	3.184	2.453	
ARG	101.D NH2	ASP	111.E OD1	2.62	1.879	
ASN	129.D ND2	SER	172.E OG	2.9	1.886	
GLN	70.E NE2	PRO	192.D O	2.984	1.974	
TRP	171.E NE1	LEU	141.D O	2.757	1.751	
EA (WT -dup)	3 H-bonds					73
SER	82.A OG	ASN	129.E OD1	3.027	2.276	
GLN	61.E NE2	SER	59.A OG	2.857	2.155	
ARG	101.E NE	TYR	83.A OH	2.509	1.595	

Table S.3 : Full list of interfacial hydrogen bond contacts in AB-dupα7 receptor before MD simulations.

Donor		Acceptor		Donor-Acceptor distance (Å)	Donor hydrogen-Acceptor distance (Å)	Total number of contacts
AB (dup-dup)	3 H-bonds					78
ARG	31.A NH1	ASP	29.B OD1	3.079	2.374	
TRP	81.B NE1	LEU	51.A O	2.806	1.972	
TYR	83.B OH	ASN	39.A OD1	2.717	1.816	
BC (dup- WT)	3 H-bonds					134
TRP	108.C NE1	GLN	13.B OE1	2.979	2.092	
SER	117.C OG	GLY	99.B O	2.766	2.064	
TRP	171.C NE1	LEU	51.B O	2.77	1.797	
CD (WT-WT)	5 H-bonds					166
LYS	28.C N	GLU	41.D OE2	3.474	2.652	
TRP	77.C NE1	ASP	119.D O	2.976	2.066	
GLN	70.D NE2	PRO	192.C O	3.166	2.201	
SER	149.D OG	GLN	61.C OE1	2.818	1.893	
TRP	171.D NE1	LEU	141.C O	2.794	1.792	
DE (WT-WT)	10 H-bonds					122
GLN	61.D NE2	SER	149.E OG	2.95	2.013	
TRP	77.D NE1	GLU	120.E OE1	2.756	1.878	
TRP	77.D NE1	GLU	120.E OE2	2.872	1.946	
ARG	101.D NH1	LYS	109.E O	3.126	2.336	
ARG	101.D NH2	ASP	111.E OD1	2.665	1.754	
ASN	129.D ND2	SER	172.E OG	2.882	1.861	
GLN	70.E NE2	PRO	192.D O	2.981	1.977	
SER	117.E OG	GLY	189.D O	2.813	2.213	
ARG	121.E NH1	PRO	143.D O	2.808	1.97	
TRP	171.E NE1	LEU	141.D O	2.803	1.801	
EA (WT -WT)	3 H-bonds					68
TRP	81.A NE1	LEU	141.E O	3.308	2.435	
SER	82.A OG	ASN	129.E OD1	3.017	2.103	
ARG	101.E NE	TYR	83.A OH	2.798	1.852	

Table S.4: Full list of interfacial hydrogen bond contacts in AC-dupα7 receptor before MD simulations.

Donor		Acceptor		Donor-Acceptor distance (Å)	Donor hydrogen-Acceptor distance (Å)	Total number of contacts
AB (dup- WT)	3 H-bonds					123
GLN	70.B NE2	PRO	102.A O	3.454	2.485	
ARG	121.B NH1	PHE	32.A O	3.199	2.306	
TRP	171.B NE1	LEU	51.A O	2.786	1.897	
BC (WT -dup)	6 H-bonds					219
ASN	75.B ND2	GLU	120.C OE1	2.686	1.819	
ARG	101.B NH1	SER	172.C O	3.267	2.418	
ASN	133.B ND2	TYR	173.C OH	3.196	2.335	
TRP	171.C NE1	LEU	141.B O	2.77	1.764	
SER	172.C OG	LEU	131.B O	3.516	3.001	
SER	172.C OG	ASN	133.B O	2.884	2.01	
CD (dup- WT)	3 H-bonds					70
ARG	101.C NH1	SER	82.D O	2.862	2.092	
SER	59.D OG	GLN	61.C OE1	3.189	2.417	
TRP	81.D NE1	LEU	141.C O	2.683	1.698	
DE (WT-WT)	4 H-bonds					177
GLN	70.E NE2	PRO	102.D O	2.883	1.878	
TRP	108.E NE1	GLN	13.D OE1	3.107	2.124	
SER	117.E OG	GLY	99.D O	2.858	2.303	
TRP	171.E NE1	LEU	51.D O	2.815	1.807	
EA (WT -dup)	3 H-bonds					62
TRP	81.A NE1	LEU	141.E O	3.267	2.39	
SER	82.A OG	ASN	129.E OD1	3.007	2.087	
ARG	101.E NE	TYR	83.A OH	2.802	1.854	

Table S.5: Full list of interfacial hydrogen bond contacts in ABC-dupα7 receptor before MD simulations.

Donor		Acceptor		Donor-Acceptor distance (Å)	Donor hydrogen-Acceptor distance (Å)	Total number of contacts
AB (dup-dup)	3 H-bonds					78
ARG	31.A NH1	ASP	29.B OD1	3.021	2.264	
TRP	81.B NE1	LEU	51.A O	2.802	1.969	
TYR	83.B OH	ASN	39.A OD1	2.711	1.803	
BC (dup-dup)	1 H-bonds					86
TRP	81.C NE1	LEU	51.B O	2.808	2.019	
CD (dup- WT)	4 H-bonds					158
GLN	70.D NE2	PRO	102.C O	3.244	2.276	
TRP	108.D NE1	GLN	13.C OE1	2.886	2.019	
TRP	171.D NE1	LEU	51.C O	2.83	1.819	
TYR	173.D OH	GLN	13.C OE1	2.956	2.251	
DE (WT-WT)	10 H-bonds					120
GLN	61.D NE2	SER	149.E OG	2.949	2.012	
TRP	77.D NE1	GLU	120.E OE1	2.763	1.887	
TRP	77.D NE1	GLU	120.E OE2	2.862	1.935	
ARG	101.D NH1	LYS	109.E O	3.13	2.34	
ARG	101.D NH2	ASP	111.E OD1	2.667	1.756	
ASN	129.D ND2	SER	172.E OG	2.887	1.866	
GLN	70.E NE2	PRO	192.D O	2.979	1.974	
SER	117.E OG	GLY	189.D O	2.81	2.207	
ARG	121.E NH1	PRO	143.D O	2.789	1.934	
TRP	171.E NE1	LEU	141.D O	2.796	1.794	
EA (WT -dup)	3 H-bonds					68
TRP	81.A NE1	LEU	141.E O	3.289	2.415	
SER	82.A OG	ASN	129.E OD1	3.014	2.1	
ARG	101.E NE	TYR	83.A OH	2.799	1.852	

Table S.6: Full list of interfacial hydrogen bond contacts in ACD-dupα7 receptor before MD simulations.

Donor		Acceptor		Donor-Acceptor distance (Å)	Donor hydrogen-Acceptor distance (Å)	Total number of contacts
AB (dup- WT)	4 H-bonds					178
GLN	70.E NE2	PRO	102.D O	2.882	1.876	
TRP	108.E NE1	GLN	13.D OE1	3.111	2.128	
SER	117.E OG	GLY	99.D O	2.854	2.295	
TRP	171.E NE1	LEU	51.D O	2.814	1.804	
BC (WT -dup)	3 H-bonds					67
TRP	81.A NE1	LEU	141.E O	3.252	2.375	
SER	82.A OG	ASN	129.E OD1	3.007	2.089	
ARG	101.E NE	TYR	83.A OH	2.801	1.853	
CD (dup-dup)	3 H-bonds					78
ARG	31.A NH1	ASP	29.B OD1	3.03	2.279	
TRP	81.B NE1	LEU	51.A O	2.801	1.967	
TYR	83.B OH	ASN	39.A OD1	2.709	1.799	
DE (dup- WT)	3 H-bonds					121
TRP	108.C NE1	GLN	13.B OE1	2.971	2.082	
SER	117.C OG	GLY	99.B O	2.766	2.059	
TRP	171.C NE1	LEU	51.B O	2.77	1.797	
EA (WT -dup)	3 H-bonds					70
ARG	101.C NH1	SER	82.D O	2.861	2.088	
SER	59.D OG	GLN	61.C OE1	3.19	2.424	
TRP	81.D NE1	LEU	141.C O	2.686	1.705	

Table S.7: Full list of interfacial hydrogen bond contacts in α7 4-dupα7 receptor before MD simulations.

Donor		Acceptor		Donor-Acceptor distance (Å)	Donor hydrogen-Acceptor distance (Å)	Total number of contacts
AB (dup - dup)	3 H-bonds					133
GLN	70.B NE2	PRO	102.A O	3.448	2.479	
ARG	121.B NH1	PHE	32.A O	3.217	2.315	
TRP	171.B NE1	LEU	51.A O	2.779	1.889	
BC (dup-dup)	5 H-bonds					149
ARG	101.B NH1	SER	82.C O	3.401	2.468	
ARG	101.B NH1	SER	87.C OG	3.029	2.241	
THR	128.B OG1	ILE	27.C O	3.459	2.578	
TRP	81.C NE1	LEU	141.B O	2.742	1.846	
TYR	83.C OH	ASN	133.B OD1	3.135	2.166	
CD (dup-dup)	3 H-bonds					76
TRP	20.D NE1	GLN	13.C OE1	2.82	1.912	
TRP	81.D NE1	LEU	51.C O	2.734	1.743	
SER	82.D OG	HIS	37.C ND1	3.087	2.181	
DE (dup- WT)	3 H-bonds					98
ARG	31.D NH1	ASP	29.E OD1	3.129	2.355	
TRP	20.E NE1	GLN	13.D OE1	3.037	2.076	
TRP	81.E NE1	LEU	51.D O	2.776	1.766	
EA (WT -dup)	4 H-bonds					89
TRP	81.A NE1	LEU	51.E O	2.789	1.925	
SER	82.A OG	ASN	39.E OD1	2.9	1.999	
THR	35.E OG1	ASP	29.A OD1	3.324	2.437	
PHE	36.E N	ASP	29.A OD1	3.274	2.411	

Table S.8: Full list of interfacial hydrogen bond contacts in 5-dupα7 receptor before MD simulations.

Donor		Acceptor		Donor-Acceptor distance (Å)	Donor hydrogen-Acceptor distance (Å)	Total number of contacts
AB (dup-dup)	3 H-bonds					77
ARG	31.A NH1	ASP	29.B OD1	2.972	2.159	
TRP	81.B NE1	LEU	51.A O	2.793	1.956	
TYR	83.B OH	ASN	39.A OD1	2.699	1.779	
BC (dup-dup)	1 H-bonds					86
TRP	81.C NE1	LEU	51.B O	2.801	2.01	
CD (dup-dup)	3 H-bonds					74
TRP	20.D NE1	GLN	13.C OE1	2.816	1.908	
TRP	81.D NE1	LEU	51.C O	2.731	1.739	
SER	82.D OG	HIS	37.C ND1	3.079	2.173	
DE (dup-dup)	3 H-bonds					99
ARG	31.D NH1	ASP	29.E OD1	3.108	2.328	
TRP	20.E NE1	GLN	13.D OE1	3.035	2.075	
TRP	81.E NE1	LEU	51.D O	2.774	1.764	
EA (dup-dup)	4 H-bonds					88
TRP	81.A NE1	LEU	51.E O	2.786	1.92	
SER	82.A OG	ASN	39.E OD1	2.894	1.981	
THR	35.E OG1	ASP	29.A OD1	3.324	2.403	
PHE	36.E N	ASP	29.A OD1	3.205	2.314	

Table S.9: Full list of interfacial hydrogen bond contacts in the canonical α7 (WT) receptor after MD simulations.

Donor		Acceptor		Donor-Acceptor distance (Å)	Donor hydrogen-Acceptor distance (Å)	Total number of contacts
AB (WT-WT)	8 H-bonds					75
ASN	75.A ND2	PHE	122.B O	2.862	1.948	
ARG	101.A NE	ASP	111.B OD1	2.821	1.85	
ARG	101.A NE	ASP	111.B OD2	2.844	1.85	
ARG	101.A NH1	LYS	109.B O	2.966	2.229	
ARG	101.A NH1	ASP	111.B OD1	2.944	2.008	
ARG	101.A NH1	ASP	111.B OD2	3.1	2.191	
ASN	133.A ND2	TYR	173.B OH	3.329	2.587	
ARG	155.B NH2	LEU	231.A O	3.322	2.344	
BC (WT-WT)	2 H-bonds					17
ARG	101.B NH2	TRP	171.C O	2.617	1.85	
THR	128.B OG1	GLU	120.C OE2	3.355	2.531	
CD (WT-WT)	1 H-bonds					105
ARG	121.D NH2	GLN	61.C OE1	3.069	2.119	
DE (WT-WT)	7 H-bonds					79
GLN	61.D NE2	ASN	69.E O	2.728	2.042	
GLN	61.D NE2	GLN	70.E O	3.536	2.589	
ASN	75.D ND2	GLU	120.E O	2.54	1.548	
ARG	101.D NH1	ASP	111.E OD1	3.162	2.199	
ARG	101.D NH1	ASP	111.E OD2	3.311	2.404	
GLN	70.E NE2	ASN	193.D O	3.56	2.883	
SER	149.E OG	GLN	61.D OE1	2.83	1.92	
EA (WT-WT)	3 H-bonds					22
ASN	46.A ND2	PRO	95.E O	3.212	2.35	
ARG	121.A NE	ASN	75.E OD1	2.833	1.884	
ARG	121.A NH1	ASN	75.E OD1	2.998	2.162	

Table S.10 : Full list of interfacial hydrogen bond contacts in A-dupα7 receptor after MD simulations.

Donor		Acceptor		Donor-Acceptor distance (Å)	Donor hydrogen-Acceptor distance (Å)	Total number of contacts
AB (dup- WT)	7 H-bonds					83
GLU	105.A N	ASN	69.B OD1	3.534	2.613	
TRP	108.B NE1	GLN	13.A OE1	2.994	2.136	
ARG	121.B NH1	PHE	32.A O	3.17	2.472	
ARG	121.B NH1	ASP	33.A OD1	2.901	2.009	
ARG	121.B NH2	ASP	33.A OD1	2.742	1.768	
ALA	124.B N	PHE	32.A O	2.877	1.908	
SER	177.B OG	GLN	11.A OE1	3.562	2.817	
BC (WT-WT)	5 H-bonds					98
LEU	29.B N	GLU	41.C OE1	3.177	2.334	
LEU	29.B N	GLU	41.C OE2	3.461	2.52	
TYR	30.B N	GLU	41.C OE1	3.523	2.519	
ARG	101.B NH1	TRP	171.C O	3.519	2.664	
SER	117.C OG	GLY	189.B O	3.519	2.807	
CD (WT-WT)	9 H-bonds					136
LYS	28.C N	GLU	41.D OE1	2.748	1.742	
LYS	28.C N	GLU	41.D OE2	3.358	2.561	
LEU	29.C N	GLU	41.D OE1	2.884	1.893	
LEU	29.C N	GLU	41.D OE2	3.23	2.475	
TYR	30.C N	GLU	41.D OE2	3.221	2.229	
ASN	75.C ND2	ASP	119.D O	3.246	2.304	
TRP	77.C NE1	ASP	119.D O	2.874	1.874	
ARG	101.C NE	SER	172.D O	2.826	2.165	
ARG	101.C NH1	SER	172.D O	2.274	1.465	
DE (WT-WT)	5 H-bonds					65
LYS	28.D N	GLU	41.E OE1	3.065	2.218	
LYS	28.D N	GLU	41.E OE2	2.995	2.078	
LEU	29.D N	GLU	41.E OE1	3.038	2.273	
ARG	101.D NH1	ASP	111.E OD2	3.359	2.536	
SER	117.E OG	SER	188.D O	3.547	2.753	
EA (WT-WT)	6 H-bonds					76
ARG	65.A NH1	ASN	193.E O	2.482	1.589	
ARG	65.A NH2	ASN	193.E O	3.193	2.52	
LYS	31.E NZ	GLN	17.A O	3.539	2.709	

ARG	101.E NE	SER	82.A OG	2.963	2.057	
ARG	101.E NH1	ASP	26.A OD1	2.642	1.92	
ARG	101.E NH1	SER	82.A OG	2.997	2.123	

Table S.11: Full list of interfacial hydrogen bond contacts in AB-dupα7 receptor after MD simulations.

Donor		Acceptor		Donor-Acceptor distance (Å)	Donor hydrogen-Acceptor distance (Å)	Total number of contacts
AB (dup-dup)	2 H-bonds					25
GLN	13.A NE2	ILE	21.B O	3.305	2.382	
ALA	23.B N	GLN	13.A OE1	3.211	2.437	
BC (dup- WT)	3 H-bonds					29
GLN	13.B NE2	TYR	173.C OH	3.062	2.378	
ARG	42.C N	GLN	13.B OE1	2.966	2.087	
TRP	171.C NE1	PRO	52.B O	2.979	2.171	
CD (WT-WT)	6 H-bonds					52
LEU	29.C N	GLU	41.D OE1	3.102	2.404	
LEU	29.C N	GLU	41.D OE2	2.689	1.978	
TYR	30.C N	GLU	41.D OE2	3.091	2.213	
GLN	106.C NE2	LEU	40.D O	3.423	2.645	
TYR	115.D OH	HIS	127.C O	2.845	2.229	
ARG	121.D NH1	GLN	61.C OE1	2.897	2.716	
DE (WT-WT)	5 H-bonds					51
LEU	29.D N	GLU	41.E OE1	2.858	1.948	
TYR	30.D N	GLU	41.E OE1	3.129	2.142	
ARG	101.D NH1	ASP	111.E OD1	2.811	1.856	
ARG	101.D NH2	ASP	111.E OD1	3.468	2.721	
ARG	101.D NH2	SER	172.E OG	3.025	2.177	
EA (WT-dup)	5 H-bonds					84
ARG	65.A NH1	GLU	195.E OE1	2.918	2.007	
ARG	65.A NH1	GLU	195.E OE2	2.894	2.02	
ARG	65.A NH2	GLU	195.E OE1	3.05	2.199	
ARG	65.A NH2	GLU	195.E OE2	2.944	2.092	
GLN	139.E NE2	SER	82.A O	3.428	2.605	

Table S.12: Full list of interfacial hydrogen bond contacts in AC-dupα7 receptor after MD simulations.

Donor		Acceptor		Donor-Acceptor distance (Å)	Donor hydrogen-Acceptor distance (Å)	Total number of contacts
AB (dup- WT)	6 H-bonds					39
GLN	11.A N	SER	172.B O	3.205	2.205	
ARG	31.A NE	PHE	122.B O	3.052	2.131	
ARG	31.A NH1	PHE	122.B O	2.893	1.961	
PHE	32.A N	ASP	123.B OD1	3.532	2.527	
GLN	49.A NE2	GLU	211.B OE2	3.546	2.627	
ALA	124.B N	PHE	32.A O	2.953	2.011	
BC (WT-dup)	1 H-bonds					117
ASN	193.B ND2	GLN	70.C OE1	3.278	2.618	
CD (dup- WT)	6 H-bonds					34
ARG	101.C NE	SER	82.D O	3.051	2.288	
ARG	101.C NH1	SER	82.D O	2.859	2.058	
ARG	121.C NH1	ASP	29.D OD2	3.104	2.106	
ARG	121.C NH2	ASP	29.D OD1	3.33	2.322	
THR	128.C OG1	ILE	21.D O	3.521	2.642	
TYR	83.D OH	ASN	129.C OD1	3.126	2.314	
DE (WT-WT)	1 H-bonds					52
ARG	42.E NE	GLN	11.D OE1	3.691	2.721	
EA (WT -dup)	1 H-bonds					58
ASN	75.E ND2	ASP	26.A O	3.475	2.476	

Table S.13: Full list of interfacial hydrogen bond contacts in ABC-dupα7 receptor after MD simulations.

Donor		Acceptor		Donor-Acceptor distance (Å)	Donor hydrogen-Acceptor distance (Å)	Total number of contacts
AB (dup-dup)	4 H-bonds					61
GLN	11.A N	SER	82.B O	3.241	2.296	
TYR	100.A N	TYR	61.B O	3.137	2.169	
TYR	61.B N	SER	98.A O	3.108	2.114	
ARG	65.B NH2	LEU	141.A O	3.354	2.411	
BC (dup-dup)	10 H-bonds					130
GLN	13.B NE2	SER	82.C O	2.8	1.83	
THR	35.B N	ASP	29.C O	2.865	1.875	
ASN	39.B ND2	ILE	21.C O	3.699	2.919	
ARG	139.B NH1	ASP	63.C OD1	2.855	1.857	
ARG	139.B NH1	ASP	63.C OD2	2.94	1.95	
ARG	139.B NH2	ASP	63.C OD1	2.568	1.752	
ARG	139.B NH2	ASP	63.C OD2	2.662	1.844	
ALA	23.C N	ASN	39.B OD1	2.95	1.968	
ASP	29.C N	THR	35.B OG1	2.892	1.907	
TYR	83.C OH	ASN	39.B OD1	2.847	1.892	
CD (dup- WT)	1 H-bonds					49
TYR	115.D OH	ASP	33.C O	2.686	1.793	
DE (WT-WT)	3 H-bonds					59
ARG	101.D NH2	ASP	111.E OD1	3.568	2.581	
ARG	101.D NH2	ASP	111.E OD2	3.544	2.569	
ARG	101.D NH2	SER	172.E OG	2.872	1.941	
EA (WT -dup)	3 H-bonds					33
TYR	83.A OH	ARG	101.E O	3.274	2.322	
LYS	28.E N	HIS	18.A O	3.528	2.843	
LYS	31.E NZ	ILE	16.A O	3.286	2.304	

Table S.14: Full list of interfacial hydrogen bond contacts in ACD-dupα7 receptor after MD simulations.

Donor		Acceptor		Donor-Acceptor distance (Å)	Donor hydrogen-Acceptor distance (Å)	Total number of contacts
AB (WT-WT)	5 H-bonds					48
GLN	13.D N	LEU	40.E O	2.97	2.016	
ARG	42.E N	GLN	11.D O	3.518	2.521	
LYS	109.E N	GLN	13.D OE1	3.223	2.261	
ARG	121.E NH1	PHE	32.D O	2.707	2.026	
ARG	121.E NH2	PHE	32.D O	3.225	2.245	
BC (WT-dup)	1 H-bonds					64
ARG	101.E NH2	SER	87.A O	2.884	2.013	
CD (dup-dup)	1 H-bonds					26
TYR	61.B OH	GLY	99.A O	3.411	2.649	
DE (dup- WT)	8 H-bonds					59
ARG	31.B NH1	GLU	120.C OE1	2.612	1.642	
ARG	31.B NH1	GLU	120.C OE2	2.662	1.658	
ARG	31.B NH2	GLU	120.C OE1	2.966	1.993	
ARG	31.B NH2	GLU	120.C OE2	2.657	1.649	
ASN	39.B ND2	TRP	171.C O	3.123	2.491	
ASN	103.B ND2	LYS	68.C O	3.102	2.536	
TRP	171.C NE1	ASN	39.B O	3.011	2.169	
LYS	214.C NZ	GLN	11.B OE1	3.012	2.202	
EA (WT -dup)	2 H-bonds					49
ASN	129.C ND2	TYR	83.D OH	2.788	1.992	
CYS	122.D SG	GLN	139.C OE1	3.595	2.308	

Table S.15: Full list of interfacial hydrogen bond contacts in 4dupα7 receptor after MD simulations.

Donor		Acceptor		Donor-Acceptor distance (Å)	Donor hydrogen-Acceptor distance (Å)	Total number of contacts
AB (dup-dup)	4 H-bonds					39
GLN	11.A N	GLU	215.B OE1	2.86	1.995	
GLN	11.A N	GLU	215.B OE2	2.817	1.922	
GLN	11.A N	TYR	217.B OH	3.318	2.494	
GLY	174.B N	GLN	13.A OE1	3.039	2.23	
BC (dup-dup)	5 H-bonds					62
THR	128.B OG1	ASP	29.C OD1	2.992	2.13	
THR	128.B OG1	ASP	29.C OD2	2.829	2.018	
ASN	129.B N	ASP	29.C OD1	3.312	2.351	
ASN	129.B N	ASP	29.C OD2	2.991	2.005	
VAL	130.B N	ASP	29.C OD2	3.362	2.355	
CD (dup-dup)	6 H-bonds					69
ARG	31.C NE	GLU	30.D OE1	2.677	1.808	
ARG	31.C NE	GLU	30.D OE2	2.689	1.822	
ARG	31.C NH1	GLU	30.D OE1	2.506	1.687	
ARG	31.C NH1	GLU	30.D OE2	2.531	1.726	
ASN	103.C N	CYS	60.D O	2.919	1.961	
SER	59.D OG	TYR	100.C O	3.182	2.29	
DE (dup-WT)	3 H-bonds					167
HIS	37.D NE2	SER	82.E OG	2.943	2.033	
ASN	39.D ND2	ILE	21.E O	3.314	2.418	
TRP	81.E NE1	LEU	51.D O	3.396	2.389	
EA (WT-dup)	3 H-bonds					138
GLY	84.A N	GLN	13.E OE1	2.992	1.992	
ALA	34.E N	ILE	27.A O	2.876	1.991	
ASN	39.E ND2	SER	82.A O	2.884	2.01	

Table S.16: Full list of interfacial hydrogen bond contacts in 5-dupα7 receptor after MD simulations.

Donor		Acceptor		Donor-Acceptor distance (Å)	Donor hydrogen-Acceptor distance (Å)	Total number of contacts
AB (dup-dup)	2 H-bonds					88
GLN	13.A NE2	TRP	81.B O	3.568	2.56	
ALA	34.A N	ILE	27.B O	3.24	2.258	
BC (dup-dup)	0 H-bonds					103
CD (dup-dup)	1 H-bonds					44
TRP	81.D NE1	HIS	37.C O	2.981	2.005	
DE (dup-dup)	1 H-bonds					106
GLU	30.E N	PHE	32.D O	2.933	1.939	
EA (dup-dup)	0 H-bonds					39

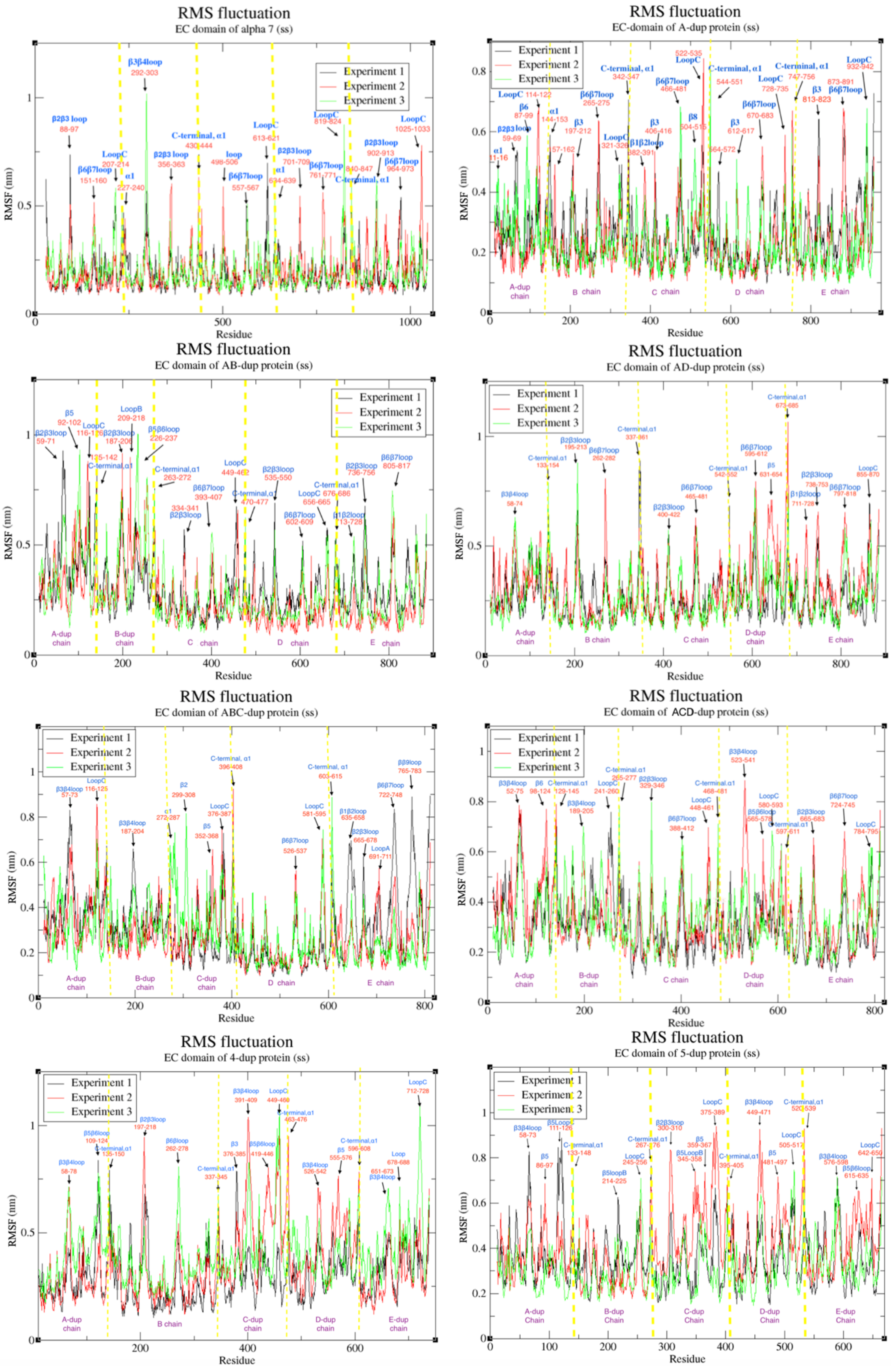

Figure S.2: A) RMSF (averaged per-residue) obtained for extracellular (EC) domains of all receptor stoichiometries investigated in this study during 100 ns of all-atom MD simulation. Each simulation has been performed in triplicate. The data for all replicas are denoted as Experiment 1 - Experiment 3.

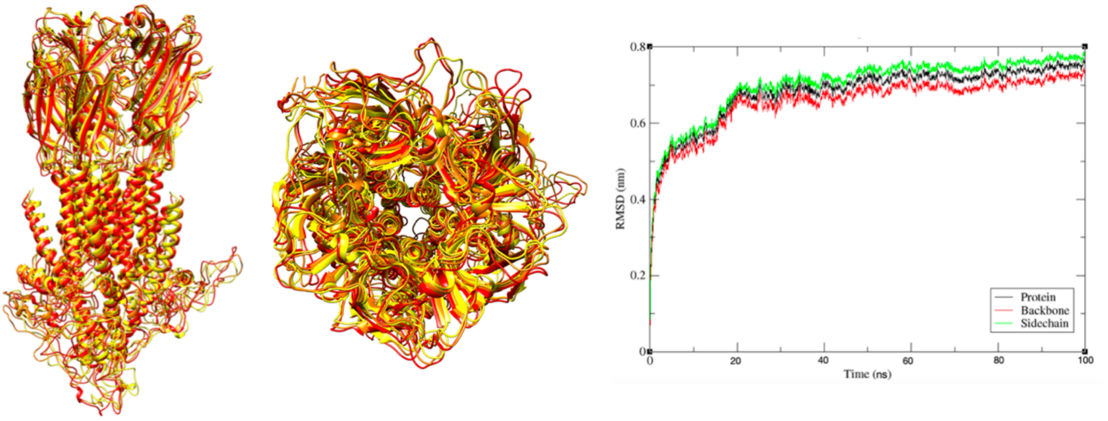

Figure S.3: Left panel: The side view of the three conformations of the full-length α7 receptor. Middle panel: The top view of the three conformations of the full-length α7 receptor. Yellow, orange and red colours signify representative configurations around 15 ns, 50 ns and 85 ns of the all-atom simulation, respectively. Right panel: root-mean-square deviation (RMSD) calculated for α7 WT receptor during 100 ns of all-atom MD simulation. Black line shows data obtained for all atoms in the full-length receptor, red line shows data for the protein backbone, green line shows data for the sidechain of α7 WT receptor.

Table S.17: Interaction between the 'gatekeeping' glutamate E254 (GLU) residue within WT-α7 and dupα7 subunits in different pentamer stoichiometries and passing Ca^{2+} cation (Ca), calculated from umbrella sampling simulations. Orange diamond represents the interaction between E254 and Ca^{2+}.

a	WT	A	AB	AD		ABC		ABD		4		5
Subunit	WT	WT	Dup	Dup	WT	Dup	Dup	Dup	WT	Dup	Dup	Dup
Ca-GLU (O)	◆											
Ca-GLU (OE1)	◆	◆	◆	◆	◆	◆		◆	◆	◆	◆	◆
Ca-GLU (OE2)	◆	◆	◆	◆	◆	◆	◆	◆	◆	◆	◆	◆
Ca-GLU (CD)	◆	◆	◆	◆	◆	◆	◆	◆	◆	◆	◆	◆
Ca-GLU (C)	◆											

Table S.18: Hydrogen bonds and electrostatic interactions between WT α7/dupα7 subunits in different receptor stoichiometries obtained from the umbrella sampling simulations. Red triangles represent the interactions between the protein residues in the canonical α7 (WT) subunit and Ca^{2+} cation. Blue diamonds represent the interactions between the protein residues in Dupα7 subunit and Ca^{2+} cation.

H-bonds	WT	A	AB	AD		ABC		ABD		4		5
Subunit	WT	WT	Dup	TW	Dup	Dup	Dup	WT	Dup	Dup	Dup	Dup
Number	6	5	5	6	5	3	5	9	8	6	6	4
LEU-PHE	▲	▲		▲	◆			▲	◆	◆		◆
VAL-PHE	▲	▲	◆	▲	◆		◆		◆		◆	
ALA-MET	▲	▲					◆		◆		◆	
GLU-LEU	▲			▲			◆	▲	◆	◆	◆	◆
ILE-LEU	▲	▲		▲		◆		▲		◆	◆	◆
MET-VAL	▲		◆	▲							◆	
VAL-MET			◆			◆		▲		◆		
TYR-ALA		▲						▲		◆		
LEU-VAL			◆	▲	◆		◆		◆		◆	
ILE-VAL			◆		◆		◆					
ALA-LEU					◆	◆		▲		◆		◆
LYS-GLU								▲				
ASN-ALA								▲				
ALA-VAL								▲				
TYR-ILE									◆			

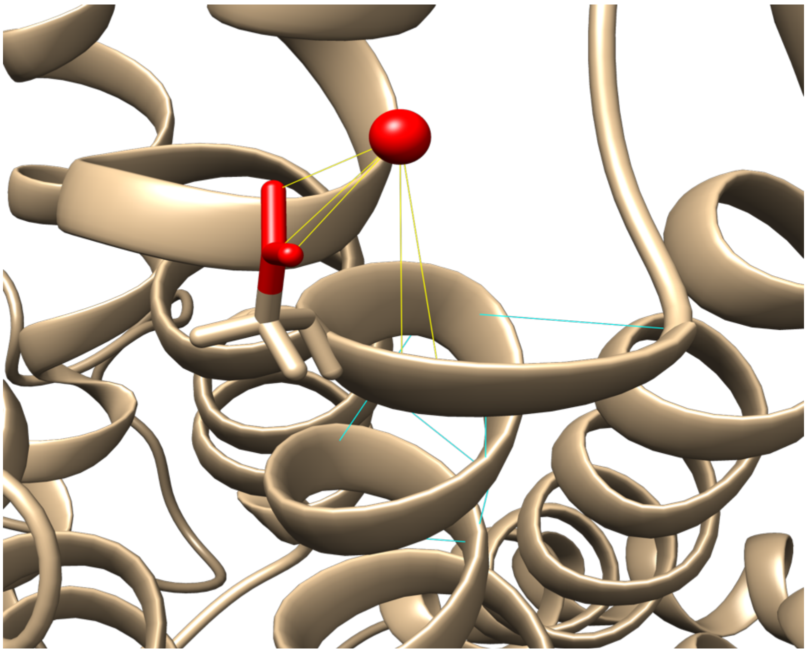

Figure S.4: The Ca^{2+} interaction scheme within the canonical (WT) receptor, at the entrance to the transmembrane (TM) region. Glutamate E254 (red) 'gatekeeper' residue interacts with the Ca^{2+} cation (yellow lines). The blue line represents the favourable electrostatic interaction between Ca^{2+} and the receptor TM region. The receptor backbone is rendered as secondary structure elements (all but E254 side chains are omitted for clarity) and coloured golden brown.
